# A high-quality genome assembly and annotation of the gray mangrove, *Avicennia marina*

**DOI:** 10.1093/g3journal/jkaa025

**Published:** 2020-12-08

**Authors:** Guillermo Friis, Joel Vizueta, Edward G Smith, David R Nelson, Basel Khraiwesh, Enas Qudeimat, Kourosh Salehi-Ashtiani, Alejandra Ortega, Alyssa Marshell, Carlos M Duarte, John A Burt

**Affiliations:** 1 Center for Genomics and Systems Biology, New York University - Abu Dhabi, PO Box 129188, Abu Dhabi, United Arab Emirates; 2 Departament de Genètica, Microbiologia i Estadística and Institut de Recerca de la Biodiversitat (IRBio), Universitat de Barcelona, Barcelona 08007, Spain; 3 Division of Biological and Environmental Sciences and Engineering, Center for Desert Agriculture, King Abdullah University of Science and Technology (KAUST), Thuwal 23955-6900, Saudi Arabia; 4 Red Sea Research Center (RSRC) and Computational Bioscience Research Center, King Abdullah University of Science and Technology (KAUST), Thuwal 23955-6900, Saudi Arabia; 5 Department of Marine Science and Fisheries, College of Agricultural and Marine Sciences, Sultan Qaboos University, Muscat, Oman

**Keywords:** gray mangrove, *Avicennia marina*, genome assembly, HiC, Arabia

## Abstract

The gray mangrove [*Avicennia marina* (Forsk.) Vierh.] is the most widely distributed mangrove species, ranging throughout the Indo-West Pacific. It presents remarkable levels of geographic variation both in phenotypic traits and habitat, often occupying extreme environments at the edges of its distribution. However, subspecific evolutionary relationships and adaptive mechanisms remain understudied, especially across populations of the West Indian Ocean. High-quality genomic resources accounting for such variability are also sparse. Here we report the first chromosome-level assembly of the genome of *A. marina*. We used a previously release draft assembly and proximity ligation libraries Chicago and Dovetail HiC for scaffolding, producing a 456,526,188-bp long genome. The largest 32 scaffolds (22.4–10.5 Mb) accounted for 98% of the genome assembly, with the remaining 2% distributed among much shorter 3,759 scaffolds (62.4–1 kb). We annotated 45,032 protein-coding genes using tissue-specific RNA-seq data in combination with *de novo* gene prediction, from which 34,442 were associated to GO terms. Genome assembly and annotated set of genes yield a 96.7% and 95.1% completeness score, respectively, when compared with the eudicots BUSCO dataset. Furthermore, an F_ST_ survey based on resequencing data successfully identified a set of candidate genes potentially involved in local adaptation and revealed patterns of adaptive variability correlating with a temperature gradient in Arabian mangrove populations. Our *A. marina* genomic assembly provides a highly valuable resource for genome evolution analysis, as well as for identifying functional genes involved in adaptive processes and speciation.

## Introduction

Mangroves are a polyphyletic group of trees and shrubs that inhabit the intertidal zone of the tropic and subtropic coasts (Nagelkerken *et al.* 2008; Polidoro *et al.* 2010; Hogarth 2015; Primavera *et al.* 2018). Mangroves share several morphological and physiological adaptations to their habitat, including traits for tolerance of high salinity, alternating dessication and submergence of soils across tidal cycles, and exposure of roots to hypoxic, sulfide-rich soils (Tomlinson 2016). Mangroves are of great ecological and economic importance, providing key functions such as high productivity, much of which is exported to surrounding ecosystems, and acting as breeding, nesting, nursery, foraging, and shelter areas for a range of biota (Nagelkerken *et al.* 2008; Lee *et al.* 2014; Carugati *et al.* 2018; Primavera *et al.* 2018). Mangroves also serve as an important carbon sink, supporting climate change mitigation and adaptation potential (Duarte *et al.* 2013). The diversity of evolutionary origins and adaptive mechanisms found in mangroves provides compelling systems for studying patterns of trait evolution, lineage divergence, and speciation (*e.g.*, Zhou *et al.* 2007; Urashi *et al.* 2013; Xu *et al.* 2017b).

Of the approximately 70 mangrove species described, the gray mangrove *Avicennia marina* has the broadest latitudinal and longitudinal distribution (Spalding *et al.* 2010; Hogarth 2015; Tomlinson 2016). At least three subspecific, partially allopatric taxa or “varieties” have been described: *A. marina* var. *australasica*, restricted to southeastern Australia and New Zealand; *A. marina* var. *eucalyptifolia*, in northern regions of Australasia; and *A. marina* var. *marina* that ranges from New Caledonia in the Pacific and across the entire Indian Ocean (Duke 1991; Li *et al.* 2016; Tomlinson 2016). The broad geographic distribution of *A. marina* is reflected in its presence across diverse environmental gradients (*e.g.*, temperature, freshwater, sediment and nutrient supply, salinity, tidal range) and spatial settings (*e.g.*, open coastlines, coastal lagoons, estuaries, deltas, coral fringes) (Duke 1990; Quisthoudt *et al.* 2012). Along with its widespread distribution, several aspects of *A. marina* biology make it a promising model organism among mangroves for the study of evolution based on genomic and molecular approaches. First, the broad environmental gradients present across the *A. marina* range are mirrored by remarkable geographic variation in phenotypic and life-history traits (Duke 1990; Tomlinson 2016), which makes them an suitable system for studying evolutionary processes related to dispersal, directional selection, and neutral evolution. Previous studies have reported the phylogenetic relationships for the varieties of *A. marina* and congeneric species (Duke *et al.* 1998; Nettel *et al.* 2008; Li *et al.* 2016), yet the specific drivers of lineage diversification remain understudied. In particular, the extensive gray mangrove populations from the eastern African and Arabian coasts have rarely been included in reported DNA sequence-based analyses, either for diversity screening or other purposes (*e.g.*, Duke *et al.* 1998; Li *et al.* 2016). Second, *A. marina* can tolerate highly variable and extreme environmental conditions, and often occupies marginal, biologically limiting environments at the edges of its distribution (Morrisey *et al.* 2010). While several biological structures and mechanisms of the gray mangrove physiology have been described (Nguyen *et al.* 2015; Naidoo 2016), the genetic basis and pathways underlying such tolerance are still largely unknown (Mehta *et al.* 2005; Jithesh *et al.* 2006; Xu *et al.* 2017a). Understudied Arabian populations represent a particular gap due to their occurrence at the extremes of air and water temperature, salinity, and aridity (Camp *et al.* 2018; Ben-Hasan and Christensen 2019). Third, the *A. marina* genome is moderately small and structurally simple compared with other eukaryotes and presents a limited amount of transposable elements (Das *et al.* 1995; Xu *et al.* 2017a; Lyu *et al.* 2018), which facilitates the identification of short polymorphisms and structural variants.

Previous studies on *A. marina* using high-throughput sequencing techniques and genomic approaches have been released. Two multispecies studies, including *A. marina*, explored patterns of convergent evolution at functional genes (Xu *et al.* 2017a) and transposable elements loads (Lyu *et al.* 2018), based on draft genomes that were recently made available online. Whole-genome assemblies of *A. marina* and several other mangrove taxa have recently been used for demographic inference (Guo *et al.* 2018) and convergent evolution analysis (He *et al.* 2020), but the underlying genomic data are not publicly accessible.

Here, we report a high-quality, chromosome-level assembly obtained using proximity ligation libraries as a resource for genome-based studies on *A. marina* and related mangrove species. A structural and functional annotation based on RNA-seq data from multiple tissues is also provided. In addition, we generated whole-genome shotgun data for a set of resequenced individuals from six different populations along the coasts of the Arabian Peninsula. We used these data to evaluate the performance of the assembly as a reference to study patterns of adaptive variability at the genomic level. Resequencing data are also used for organelle assembling.

## Materials and methods

### Genome sequencing and assembly

**Figure 1:**
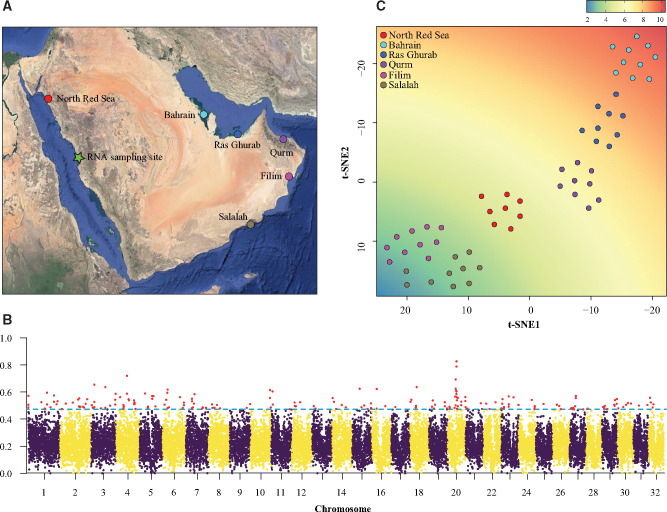
Geography and adaptive variability in Arabian gray mangroves. (A) Locations of the six stands sampled for whole-genome resequencing (colored circles) and for RNA-seq (green star). (B) F_ST_ genome scan based on 22,181 windows of 20 kb. Boxplot outliers (coefficient = 1.5) are marked in red (C) t-SNE based on 613 SNP outliers linked to functional genes. The background shows the correlation of t-SNE1 and t-SNE2 with the annual temperature range registered in each one of the sampling locations. Temperature depicted in the legend is in °C.

A high-quality genome was produced combining a previously released draft assembly of *A. marina* var. *marina* (GenBank assembly accession: GCA_900003535.1, GCA_900174615.1; Xu *et al.* 2017a; Lyu *et al.* 2018) and newly generated sequence data from proximity ligation libraries. Preparation of proximity ligation libraries Chicago and HiC as well as scaffolding with the software pipeline HiRise (Putnam *et al.* 2016; https://dovetailgenomics.com) was conducted at Dovetail Genomics, LLC. The sequenced sample was leaf tissue obtained from an individual of *A. marina* var. *marina*, located at Ras Ghurab Island in the Arabian Gulf (Figure 1; Abu Dhabi, United Arab Emirates; 24.601°N, 4.566°E). Briefly, for Chicago and Dovetail HiC libraries preparation, chromatin was fixed with formaldehyde. Fixed chromatin was then digested with DpnII and free blunt ends were ligated. Crosslinks were reversed, and the DNA purified from protein, which was then sheared to ∼350 bp mean fragment size. Libraries were generated using NEBNext Ultra enzymes and Illumina-compatible adapters, and sequencing was carried out on an Illumina HiSeq X platform. Chicago and Dovetail HiC library reads were then used as input data for genome assembly for HiRise, a software pipeline designed specifically for using proximity ligation data for high-level scaffolding of draft genomes (Supplementary Figures S1 and S2; Putnam *et al.* 2016). The previously reported draft genome of *A. marina* was used in the assembly pipeline, excluding scaffolds shorter than 1 kb since HiRise does not assemble them. Further details are provided in the Supplementary information.

The mitochondrial genome was assembled using NOVOplasty2.7.2 (Dierckxsens *et al.* 2017) and resequencing data based on Illumina paired-end 150-bp libraries from a conspecific individual (see below; Supplementary information). The maturase (matR) mitochondrial gene available in NCBI (GenBank accession no. AY289666.1) was used for the input seed sequence. The assembly of the chloroplast yielded a high number of alternative, unsolved assemblies and is thereby no reported.

### Genome annotation

We performed the annotation of the *A. marina* genome using mRNA data from a set of tissues of conspecific individuals in combination with *de novo* gene prediction using BRAKER2 v2.1.5 (Hoff *et al.* 2016). Samples were collected on the coast of the Eastern Central Red Sea north of Jeddah in the Kingdom of Saudi Arabia (22.324°N, 39.100°E; Figure 1A). Total RNA was isolated from root, stem, leaf, flower, and seed using TRIzol reagent (Invitrogen, USA). RNA-seq libraries were prepared using TruSeq RNA sample prep kit (Illumina, Inc.), with inserts that range in size from approximately 100–400 bp. Library quality control and quantification were performed with a Bioanalyzer Chip DNA 1000 series II (Agilent) and sequenced in an HiSeq2000 platform (Illumina, Inc.). First, repetitive regions were modeled *ab initio* using RepeatModeler v2.0.1 (Flynn *et al.* 2019) in all scaffolds longer than 100 kb with default options. The resulting repeat library was used to annotate and soft-mask repeats in the genome assembly with RepeatMasker 4.0.9 (Smit *et al.* 2015). Next, messenger RNA reads were mapped against the soft-masked genome assembly with HISAT2 (Kim *et al.* 2015). Gene prediction was conducted with BRAKER2 using both the RNA-seq data and the conserved orthologous genes from BUSCO Eudicots_odb10 as proteins from short evolutionary distance to provide hints and train GeneMark-ETP and Augustus (–etpmode; Lomsadze *et al.* 2005; Stanke *et al.* 2006; Gotoh 2008; Stanke *et al.* 2008; Li *et al.* 2009; Barnett *et al.* 2011; Iwata and Gotoh 2012; Lomsadze *et al.* 2014; Buchfink *et al.* 2015; Hoff *et al.* 2019; Bruna *et al.* 2020). The obtained gene annotation gff3 file was validated and used to generate the reported gene annotation statistics with GenomeTools (Gremme *et al.* 2013) and in-house Perl scripts. Finally, we conducted a similarity-based approach to assist the functional annotation of the predicted proteins. We integrated InterProScan v5.31 (Jones *et al.* 2014) and BLAST (Tatusova and Madden 1999) searches using the UniProt Swiss-Prot database and the annotated proteins from the *Arabidopsis thaliana* genome (UniProt Consortium 2019) to generate a final set of annotated functional genes (Supplementary Figure S4). Further details on mRNA sequencing and annotation scripts are provided in the Supplementary information.

### Gene completeness assessment

We assessed gene completeness in the genome assembly, and gene annotation, using BUSCO (Benchmarking Universal Single-Copy Orthologs) v4.0.5 (–auto-lineage-euk option; Waterhouse *et al.* 2018). BUSCO evaluations were conducted using the 255 and 2326 single-copy orthologous genes in Eukaryota_odb10 and Eudicots_odb10 datasets, respectively.

### *Adaptive variability analysis and functional assessment of* A. marina *genome*

To test the potential of the assembly and annotation reported here as a resource for genomic-based studies, we checked for regions of high divergence across the genome of *A. marina* using newly generated whole-genome data. We resequenced 60 individuals from six different sampling sites from each of the major seas bordering Arabia (Figure 1A; Supplementary Table S1), including populations in the Red Sea, the Arabian Gulf, and the Arabian Sea/Sea of Oman. Arabia’s regional seas present extreme, but divergent, environmental conditions for mangrove growth. The northern Red Sea is characterized by colder winter temperatures and high salinity (Carvalho *et al.* 2019), while the southern Persian/Arabian Gulf is the world’s hottest sea each summer and is also hypersaline, with both areas considered arid to hyperarid with limited (<200 mm) rainfall (Vaughan *et al.* 2019). In contrast, the Arabian Sea and Sea of Oman have normal oceanic salinity, and summer temperatures that are buffered by cold-water upwelling as a result of the Indian Ocean monsoon, resulting in more benign environmental conditions (Burt *et al*. 2016; Claereboudt 2019). Using our new *A. marina* genome assembly as a reference, we mapped the resequenced data with the mem algorithm implemented in the Burrows-Wheeler Aligner (BWA; Li and Durbin 2009) and conducted the variant calling with the Genome Analysis Toolkit (GATK; McKenna *et al.* 2010). Applying heavy quality filters, a matrix of genome-wide SNPs was generated. Using vcftools (Danecek *et al.* 2011), we computed Weir and Cockerham (W&C; Weir and Cockerham 1984) Weighted F_ST_ values based on 20-kb sliding windows, and defining each sampling site as a population to be compared. Based on the distribution of F_ST_ values, boxplot outlier loci (coefficient = 1.5) associated with functional genes were identified. We then used these loci to explore geographic patterns of adaptive variability by means of t-SNE analysis, testing for correlations between variability in sea surface temperature (SST) and t-SNE scores. Details on sequencing, variant calling, and analytical procedures for this section are available in the Supplementary information.

### Data availability

Genome assembly, genome annotation, and related supporting resources for facilitating the use of these data have been deposited at DRYAD (doi:10.5061/dryad.3j9kd51f5). SNP matrix from resequenced individuals used in adaptive variation analyses is also provided in this dataset in vcf format. Raw resequencing data have been deposited in GenBank under the accessions SRA: SRP265707; BioProject: PRJNA629068.

The genome assembly has also been deposited at DDBJ/ENA/GenBank along with raw sequence data from Chicago and HiC libraries under the accession JABGBM000000000, SRA: SRP265707; BioProject: PRJNA629068.

Datasets relating to the RNA-seq analysis have been deposited in Mendeley (doi:10.17632/9tsp7fr28r). The RNA-seq reads have been deposited at GenBank under the Bioproject (SRA) accession: PRJNA591919; Biosample accession: SAMN13391520.

Supplementary material is available at figshare DOI: https://doi.org/10.25387/g3.13270154.

## Results and discussion

### Sequencing and genome assembly

**Figure 2:**
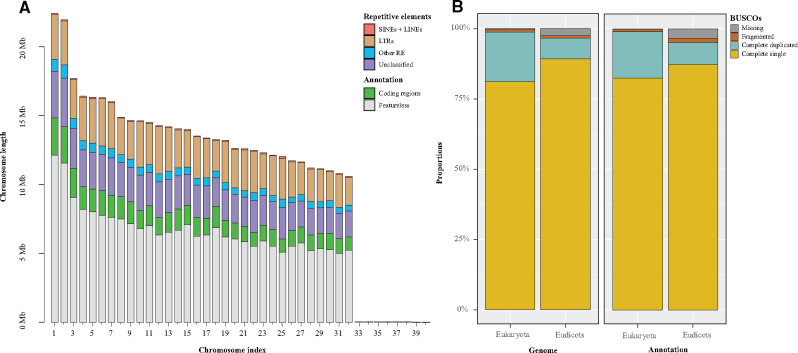
High-quality assembly and annotation for *A. marina*. (A) Length bar-plot of the longest 40 scaffolds arranged by decreasing size. Bars show per-chromosome genomic proportions corresponding to identified gene regions; and repetitive elements (SINEs + LINEs: short and long interspersed nuclear elements; LTRs, long terminal repeats). (B) BUSCO completeness percentages using the eukaryota (*N* = 255) and eudicots (*N* = 2,326) databases for the genome assembly (left) and for the annotation (right).

We sequenced and assembled a reference genome of the gray mangrove. Chicago and Dovetail HiC libraries produced 235 and 212 million 2 × 150 bp paired-end reads, respectively, and 134.1 Gb data overall. Genome scaffolding with HiRise yielded an assembly of 456.5 Mb; an L50/N50 equal to 15 scaffolds/13.98 Mb and an L90/N90 of 29 scaffolds/11.14 Mb; and a relatively large number of ambiguous bases (*i.e.*, N) inserted in the genome (10.6%; Table 1). The scaffold length distribution was heavily skewed toward extreme values (Table 1, Figure 2A). The 32 longest scaffolds, ranging from 22.40 to 10.58 Mb (median = 13.44 Mb) accounted for 98.03% of the whole assembled genome, congruent with a chromosome number 2N = 64 reported earlier (He *et al.* 2020). The remaining 1.97% of the genome was distributed among another 3,759 scaffolds ranging from 62.5 kb to over 1 kb (median = 1.8 kb; Figure 2A). The large number of small scaffolds may be due to the high fragmentation of the draft genome used in the assembly pipeline (Dovetail, personal communication). The integrity assessment of the *A. marina* genome retrieved a 98.8% and a 96.7% of the tested BUSCO groups for the eukaryote and the eudicots databases, respectively (Table 1, Figure 2B). The remarkable discontinuity in length sizes, as well as the integrity and high quality of the scaffolding lends considerable support to the hypothesis of 32 chromosomes; further sequencing efforts involving long-read sequencing are warranted for confirmation. The mitochondrial genome assembly was 22,019 pb long with a 46.4% GC content.

**Table 1:** Summary statistics for the genome assembly and annotation of *A. marina*

Genome assembly	
Total length	456,526,188 bp
Number of scaffolds	3,791
N50/L50	13,979,447 bp/15 scaffolds
N90/L90	11,144,373 bp/29 scaffolds
Chromosome scale	10,583,658 bp/32 scaffolds
Longest scaffold	22,400,447 bp
Missingness	10.6%
GC content	35.2 %
BUSCO eukaryota database	C: 98.8% [S: 81.2%, D: 17.6%], F: 0.8%, M: 0.4%, *N* = 255
BUSCO eudicots database	C: 96.7% [S: 89.2%, D: 7.5%], F: 0.8%, M: 2.5%, *N* = 2,326

**Genome annotation**	

Number of genes	41,206
Number of annotated genes	35,604
Number of genes with GOs	34,442
Average gene length	3,152.28
Number of CDS	45,032
Average CDS length (bp)	1,097.74
Number of exons	233,312
Average exon length (bp)	211.87
Number of introns	188,280
Average intron length (bp)	536.98
BUSCO eukaryota database	C: 98.9% [S: 82.4%, D: 16.5%], F: 0.8%, M: 0.3%, *N* = 255
BUSCO eudicots database	C: 95.1% [S: 87.3%, D: 7.8%], F: 1.4%, M: 3.5%, *N* = 2,326

BUSCO parameters are C, complete BUSCO; S, complete and single-copy BUSCOs; D, complete and duplicated BUSCOs; F, fragmented BUSCOs; M, missing BUSCOs; *N*, total BUSCO groups searched. CDS indicates protein-coding sequences.

### Genome annotation

We identified 45,032 protein-coding sequenced for which 35,604 showed homology with proteins from other species, and 34,442 were associated to GO (gene ontology) terms. The average gene length was 3.15 kb, with a mean of 5.2 exons and 4.2 introns per gene. BUSCO integrity analysis reported a 98.9% of recovered complete BUSCOs for the eukaryota database, and a 95.1% in the case of the eudicots (Table 1, Figure 2B). We also found that a total of 40.2% (188.5 Mb) of the *A. marina* assembly consisted of repetitive elements, a value moderately larger than the 30.4% previously reported for the species (Xu *et al.* 2017a). The greatest proportions corresponded to long terminal repeats and unclassified elements (20% and 16.7%, respectively; Figure 2A; Supplementary Table S2).

### *Adaptive variability analysis and functional assessment of* A. marina *genome*

We resequenced 60 individuals of *A. marina* from six different populations across the environmentally diverse coasts of the Arabian Peninsula (Figure 1A, Supplementary Table S1) with a coverage of 85X. After SNP calling and a strict filtering for quality and missing data, we obtained a dataset of 538,185 SNPs for 56 individuals. A W&C Weighted F_ST_ scan based on sliding 20-kb windows revealed a heterogeneous landscape of differentiation and detected a peak of high divergence at the Scaffold 20 (Figure 1B). A total of 200 highly divergent loci were identified, from which 123 (61.5%) overlapped with 109 annotated genes associated to GO terms (Supplementary Table S3 and Figure S4). Importantly, we found signals of differentiation in genes involved in regulatory networks and biological pathways related to salinity stress response (*HDA19*, *NHL6*, and *ASG2*; Chen and Wu 2010; Bao *et al.* 2016; Dutilleul *et al.* 2016; Ueda *et al.* 2017); drought resistance and stomatal conductance (*NPK1* and *NCED*; Ning *et al.* 2008; Speirs *et al.* 2013); leaf cuticle development (*GPAT4*; Fabre *et al.* 2016); response to heat stress (*IDM1*; Zhao *et al.* 2014); sensitiveness to UV-B radiation (*SAD2*; Chen *et al.* 2013) and red light (*GIL1*; Allen *et al.* 2006); as well as other responses to abiotic stressors including root development and homeostasis maintenance (*CTL1*; Hermans *et al.* 2010; Gao *et al.* 2017), and inhibition of responses to osmotic stress by means of chromatin condesantion (*SLK2*; Shrestha *et al.* 2014), supporting the role of environmental, divergent selective pressures in the differentiation of Arabian mangroves (Supplementary Table S3). A t-SNE based on 613 SNPs extracted from the functionally annotated, highly divergent loci identified in the F_ST_ scan showed clear clustering patterns among sampled populations (Figure 1C). Loading scores of retained t-SNE axes showed high correlation with the gradient of SST (*P*-values below 2.0 × 10^−16^ for both t-SNE1 and t-SNE2), also congruent with a differentiation process driven by environmental factors. In light of these results, further research into gene evolution and biological pathways involved in local adaptation to the extreme environment of the Arabian mangroves is in process. These questions are, however, beyond the scope of this report and thus will be presented elsewhere.

In conclusion, we report the first chromosome-scale assembly for the *A. marina* genome along with a comprehensive annotation based on tissue-specific RNA-seq data. The genome is highly contiguous and complete, and we demonstrated that it is a valuable resource for variant calling and the identification of functional, candidate genes underlying phenotypic and environmental divergence among mangrove taxa. Moreover, the successful identification of candidate genes potentially involved in local adaptation reveals the genome and annotation here reported as a relevant tool for the identification of biological pathways underlying molecular responses to extreme environmental conditions, which can in turn help to characterize adaptive mechanisms at the transcriptional and metabolomic levels. Improved scaffolding also enables the identification of regions putatively under selection, including structural variants such as chromosome rearrangements or copy number variations, all relevant for investigating questions related to evolutionary biology and molecular ecology in this ecological and socioeconomically important species.
